# Trade, investment and nutrition: lessons from the Canadian case for food systems governance

**DOI:** 10.1017/S1368980026102134

**Published:** 2026-02-19

**Authors:** Sylvain Charlebois

**Affiliations:** Agri-Food Analytics Lab, Dalhousie University, 6100 University Boulevard, Halifax, NS B2X 3T5, Canada

**Keywords:** Trades and investment, Health outcomes, Measurements, Canada

This commentary responds to the study by Kelly Garton *et al.*, ‘Monitoring the impacts of international trade and investment agreements on food environments: a Canadian case study’, published in Public Health Nutrition, which applies the INFORMAS trade and investment monitoring framework to the Canadian food system.

International trade and investment agreements (TIAs) have become central yet often underappreciated determinants of modern food systems. While nutrition policy debates frequently focus on consumer behaviour, dietary guidelines or retail food prices, the macroeconomic rules governing how food is produced, processed, traded and invested in shape the boundaries within which these downstream outcomes emerge. A growing body of work situates TIA firmly within the commercial determinants of health framework, highlighting their influence on dietary patterns and population health outcomes^(1–3)^. In this context, the Canadian case study applying the INFORMAS trade and investment monitoring protocol entitled ‘Monitoring the impacts of international trade and investment agreements on food environments: a Canadian case study’ provides a timely and methodologically rigorous contribution to public health nutrition.

By examining tariffs, trade flows, foreign direct investment (FDI) and trade-related policy space over nearly three decades, the study advances understanding of how trade governance structures intersect with national food environments. Monitoring approaches of this kind have previously been applied only in a limited number of small or middle-income countries, making this large, high-income case particularly valuable for international comparison^(1,4)^.

One of the study’s most important contributions lies in its historical perspective. The authors demonstrate that the most substantial tariff reductions affecting Canada’s food system occurred around the country’s accession to the World Trade Organization in 1995, with limited subsequent change outside supply-managed sectors such as dairy and eggs. This finding underscores the path-dependent nature of trade policy and reinforces evidence that contemporary food environments are shaped largely by historic liberalisation decisions rather than recent trade agreements^(5–7)^.

From a public health nutrition standpoint, this challenges a common narrative that attributes current dietary challenges primarily to recent trade deals. Instead, the Canadian experience suggests that trade liberalisation has long been ‘locked in’ and that current food environments reflect structural conditions established in the late twentieth century. International evidence similarly shows that World Trade Organization accession is associated with persistent increases in energy availability, dietary risk factors and processed food supply that extend well beyond the initial liberalisation period^(5,8)^.

The study also makes an important conceptual contribution by highlighting the nutrition-neutral design of TIA. Canadian tariff schedules and trade rules do not explicitly differentiate foods based on nutritional quality or degree of processing. At face value, this neutrality might suggest that trade policy is largely irrelevant to nutrition outcomes. However, the empirical findings challenge this assumption. While trade openness applies broadly across food categories, the benefits of liberalisation are not evenly distributed across producers or products^(2,9)^.

Ultra-processed foods, as defined by the NOVA classification system, appear particularly well positioned to capitalise on trade and investment liberalisation. Their long shelf life, standardised formulations and compatibility with global branding and marketing strategies give them structural advantages in international markets. The Canadian data show sustained growth in both imports and exports of processed and ultra-processed food categories, including sugars, soft drinks, ready meals and sauces. This pattern aligns closely with international findings linking trade openness and foreign investment to increased availability and consumption of ultra-processed foods^(9–11)^.

Crucially, the study does not argue that TIA explicitly promote unhealthy foods. Rather, it demonstrates that nutrition-neutral rules interact with market structures in ways that favour large, capital-intensive firms capable of operating at scale. Smaller producers of fresh or minimally processed foods often face higher relative barriers related to perishability, compliance costs and logistics. As a result, trade liberalisation may inadvertently amplify existing imbalances within food systems, reinforcing dietary patterns that public health policy seeks to moderate^(3,12)^.

FDI trends reinforce this concern. The authors show that FDI into Canada’s food manufacturing sector has grown steadily since the mid-2000s, reaching over CAD 30 billion in recent years. This level of investment far exceeds flows into primary agriculture or food services, underscoring the central role of food processing and manufacturing in contemporary food systems. Similar patterns have been documented globally, where FDI concentrates in sectors characterised by high degrees of processing and brand value^(13,14)^.

From a public health perspective, rising FDI and corporate concentration raise important questions about market power, product formulation and consumer choice. High levels of concentration have been observed in several ultra-processed food categories in Canada and internationally and are increasingly linked to aggressive marketing strategies and reduced incentives for nutritional reformulation^(14,15)^.

Perhaps the most policy-relevant dimension of the study concerns regulatory space. While Canada has not been the respondent in formal trade disputes over healthy food environment policies, the authors document Canada’s active participation in raising specific trade concerns at the World Trade Organization related to nutrition labelling measures in other countries. In several cases, these concerns were associated with delayed implementation, policy modification or dilution of public health measures^(1,16)^.

This finding is consistent with the concept of regulatory chill, whereby the threat or anticipation of trade challenges influences policy design and ambition. Importantly, regulatory chill does not require formal dispute settlement to be effective. The costs of defending innovative nutrition policies in international forums can be substantial, particularly for low- and middle-income countries with limited legal and technical capacity^(17,18)^.

The Canadian case is instructive because it highlights how countries with strong domestic public health commitments may nonetheless contribute to global governance dynamics that constrain nutrition policy elsewhere. This raises important questions about coherence between national health objectives and international trade diplomacy, particularly as nutrition labelling, marketing restrictions and fiscal measures become central tools for addressing diet-related noncommunicable diseases^(16,19)^.

Methodologically, the study demonstrates the value of systematic monitoring approaches such as INFORMAS. While limitations related to data availability and food classification systems remain, monitoring does not seek to establish causality. Instead, it provides essential contextual evidence to inform anticipatory governance and policy design, particularly in complex domains where experimental evaluation is infeasible^(1,4)^.

Looking forward, several priorities emerge for research and policy. Greater integration of trade, nutrition and competition policy analysis is needed to understand how market structures mediate the effects of liberalisation on food environments. Monitoring frameworks could be expanded to incorporate nutrient composition, affordability and marketing exposure, complementing existing trade indicators. Health impact assessments of TIA should also move from aspirational commitments to routine practice.

In conclusion, the Canadian case study offers a nuanced and empirically grounded account of how TIA shape food environments over time. It challenges simplistic narratives about trade and nutrition, highlights structural asymmetries embedded in ostensibly neutral rules and draws attention to the often-overlooked role of policy space in shaping nutrition outcomes. For public health nutrition scholars and policymakers, the study reinforces the importance of engaging with trade governance as a core component of food systems transformation.
